# Revealing Topological Organization of Human Brain Functional Networks with Resting-State Functional near Infrared Spectroscopy

**DOI:** 10.1371/journal.pone.0045771

**Published:** 2012-09-24

**Authors:** Haijing Niu, Jinhui Wang, Tengda Zhao, Ni Shu, Yong He

**Affiliations:** State Key Laboratory of Cognitive Neuroscience and Learning, Beijing Normal University, Beijing, People’s Republic of China; Wake Forest School of Medicine, United States of America

## Abstract

**Background:**

The human brain is a highly complex system that can be represented as a structurally interconnected and functionally synchronized network, which assures both the segregation and integration of information processing. Recent studies have demonstrated that a variety of neuroimaging and neurophysiological techniques such as functional magnetic resonance imaging (MRI), diffusion MRI and electroencephalography/magnetoencephalography can be employed to explore the topological organization of human brain networks. However, little is known about whether functional near infrared spectroscopy (fNIRS), a relatively new optical imaging technology, can be used to map functional connectome of the human brain and reveal meaningful and reproducible topological characteristics.

**Results:**

We utilized resting-state fNIRS (R-fNIRS) to investigate the topological organization of human brain functional networks in 15 healthy adults. Brain networks were constructed by thresholding the temporal correlation matrices of 46 channels and analyzed using graph-theory approaches. We found that the functional brain network derived from R-fNIRS data had efficient small-world properties, significant hierarchical modular structure and highly connected hubs. These results were highly reproducible both across participants and over time and were consistent with previous findings based on other functional imaging techniques.

**Conclusions:**

Our results confirmed the feasibility and validity of using graph-theory approaches in conjunction with optical imaging techniques to explore the topological organization of human brain networks. These results may expand a methodological framework for utilizing fNIRS to study functional network changes that occur in association with development, aging and neurological and psychiatric disorders.

## Introduction

The human brain is a highly complex network that is interconnected structurally by a dense of cortico-cortical axonal pathways [1] and functionally through synchronized or coherent neural activity [2]. Mapping the human connectome and highlighting its underlying organizational principles are crucial to understanding the architecture of the brain and revealing connectivity changes in entire assemblages of the brain that occur in response to neurological and psychiatric disorders.

Recent studies have shown that human brain networks can be constructed from a variety of neuroimaging and neurophysiological techniques (e.g., structural MRI, diffusion MRI, functional MRI and electroencephalography/magnetoencephalography) and further quantitatively analyzed with graph-theory methods. Graph-based network analysis approaches are straightforward but powerful in characterizing topological properties of the brain networks. Using this theory, it has been shown that human brain networks possess many non-trivial topological properties such as small-world topology, modularity and highly connected hubs [3]–[6]. Moreover, these properties exhibit specific alterations during normal development, aging or under pathological conditions [7]–[11]. Although several imaging techniques have been employed extensively to study connectivity patterns in the brain, it is still largely unknown whether functional near infrared spectroscopy (fNIRS), a relatively new optical imaging technology, can be used to map the functional connectome of the human brain and reveal its underlying infrastructure.

The fNIRS technique uses light in the near-infrared spectrum (670–900 nm) to noninvasively monitor hemodynamic responses evoked by brain activity and to obtain quantitative concentration changes in two chromophores of oxygenated hemoglobin (oxy-Hb) and deoxygenated hemoglobin (deoxy-Hb) in blood flow [12], [13]. Relative to functional MRI (fMRI), fNIRS has several advantages such as portability, a lower cost, and a higher temporal sampling rate (≥10 Hz). It is also more convenient for studying special populations (e.g., infants and patients with severe movement disorders). To date, fNIRS has been increasingly used not only to localize focal brain activation during cognitive engagement [14]–[19], but also to map the functional connectivity of spontaneous brain activity during resting state [20]–[23]. The resting state is a natural condition in which there is neither overt perceptual input nor behavioral output. Due to its convenience and comparability across different studies and its reflection of spontaneous brain activity, the resting state is becoming a vital experimental paradigm to study brain function [24], [25]. There are currently two strategies for deriving resting-state functional connectivity from fNIRS data: one is a seed-based correlation approach that computes temporal correlations between a pre-defined channel of interest and other channels, and the other is independent component analysis (ICA) which utilizes the whole dataset (i.e., all channels) to divide the brain into several statistically independent functional systems (i.e., components). Using these two approaches, several studies have demonstrated strong functional connectivity between bilateral sensorimotor, auditory and visual systems in adults [20]–[22] and connectivity changes during the normal development of early infancy [26] and in neurological disorders [27]. Importantly, the resting-state functional connectivity revealed by fNIRS has also been proven to be test-retest reliable at both individual and group levels [28] and reproducible among various imaging systems [29]. Therefore, these studies have provided evidence that fNIRS has the power to detect the functional connectivity of the brain. However, it should be noted that the current fNIRS analysis methods (e.g., seed- or ICA-based functional connectivity) can only be used to reveal single functional connections or connectivity components of the brain. They cannot uncover organizational principles governing these connectivity patterns.

In this study, we aim to use resting-state fNIRS (R-fNIRS) and graph-theory methods to investigate the topological architecture of functional connectivity patterns in the human brain. The motivation of the current study is that if R-fNIRS can be used successfully to map brain connectome and reveal reproducible and meaningful topological architecture, it will not only broaden our understanding of functional brain connectome but also expand methodological framework for current connectome studies. This is extremely attractive given several unique advantages of fNIRS, such as high temporal resolution and insensitivity to subject motion, which enable researchers to exploit dynamically instantaneous changes of functional brain connectome and special populations (e.g., neonates). To this end, we collected R-fNIRS data of 15 healthy young adults and then constructed brain functional networks by computing correlation matrices between the time series of 46 measurement channels for each participant. The resulting correlation matrices were then averaged to obtain a population-based connectivity backbone network. Finally, we calculated several topological parameters (e.g., small-world, efficiency, module and network hubs) of the group-level brain network as a function of connectivity thresholds and further examined the reproducibility of our findings. This allows us to utilize R-fNIRS data and graph-theory methods to systematically investigate the topological organization of human brain functional networks.

## Materials and Methods

### Participants and Protocol

Participants included 21 healthy young adults who were between 18 and 26 years of age (15 male, mean age 23.5 years). During R-fNIRS data collection, the participants were instructed to remain still and keep their eyes closed without falling asleep. For each participant, the data collection lasted 10 minutes. We excluded data from 6 participants because of large motion artifacts in the signals due to head movements or because of failure in probe placement due to obstruction by hair (see Data preprocessing). Thus, only data from 15 participants (10 male, mean age 22.3 years) were included in the final analysis. All participants provided written informed consent, and this study was approved by the Institutional Review Board of the State Key Laboratory of Cognitive Neuroscience and Learning at Beijing Normal University.

### Data Acquisition

**Figure 1:**
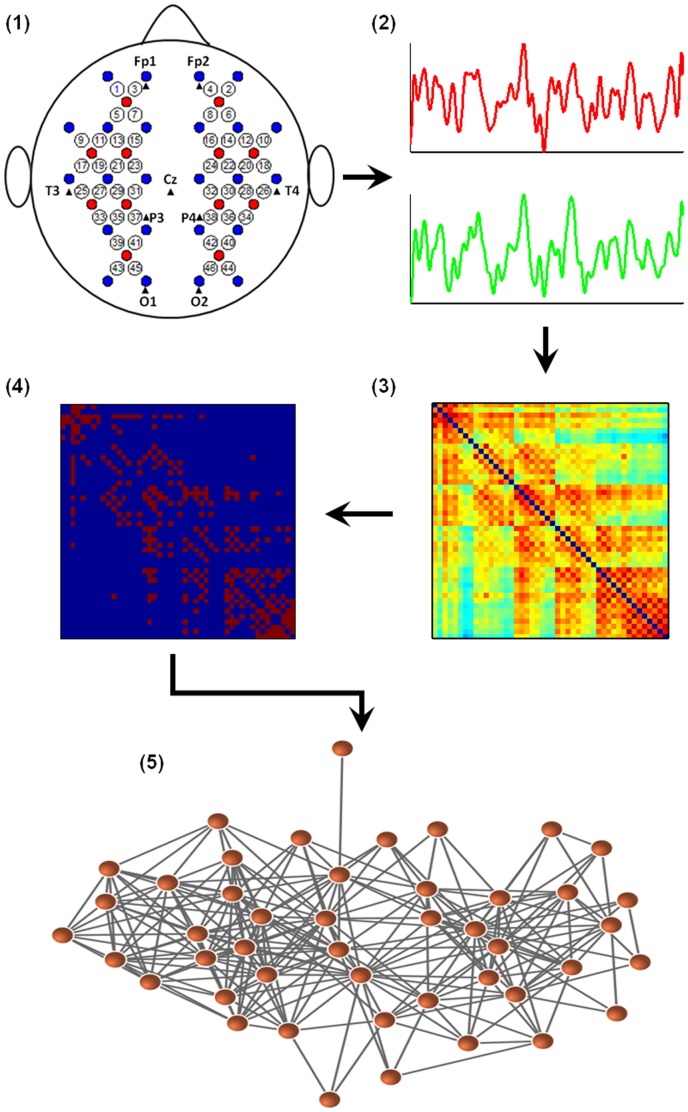
Flowchart for the construction of a functional brain network using R-fNIRS data. (1) Schematic arrangement of the probe array (12 sources, red, and 24 detectors, blue, which configurate 46 measurement channels over the whole head, as indicated by digits from 1 to 46). (2) Extraction of the time course from R-fNIRS data from each measurement channel (i.e., network node). (3) Calculation of the correlation matrix for all pairs of channels or nodes. (4) Thresholding of the correlation matrix into a binary adjacency matrix. (5) Visualization of the binary adjacency matrix as a graph.

A continuous wave (CW), near-infrared diffuse optical tomography instrument (CW6, TechEn Inc., MA, USA) was used for data acquisition. The instrument generated two wavelengths of near-infrared light (690 and 830 nm) and measured the time courses of changes in oxyhemoglobin (oxy-Hb) and deoxyhemoglobin (deoxy-Hb) for multiple channels based on the modified Beer-Lambert law [30]. The instrument consisted of 12 laser sources (each with two wavelengths) and 24 detectors. During the experiment, these sources and detectors were systematically embedded in a soft plastic holder that was then secured to the participant’s head with Velcro straps. Each adjacent source-detector pair defined a single measurement channel, to be set at 3.2 cm on the spatial separation. This design allowed for 46 different measurement channels, and guaranteed that almost the whole head, including frontal, temporal, parietal and occipital lobes of each hemisphere would be covered (Fig. 1A). The positioning of the probe array was determined according to the international 10–20 system of electrode placement and referred to the external auditory canals and vertex of each participant as landmarks. Specifically, the detectors below channels 17 to 24 in both hemispheres were set along a coronal line from the vertex to the external auditory pores, thus their midline was localized in Cz and the leftmost and rightmost detectors were fitted around T3 and T4, respectively. The position of the array relative to the landmarks was measured to establish repeatable positioning.

### Data Preprocessing

We excluded the data that included motion artifacts by examining visually sharp changes in the time series of hemoglobin concentration [27], [31]–[33]. We also used visual inspection to remove data that contained low signal-to-noise ratio at one or several channel(s) due to failures in source/detector placement [21]. With these strict criterions, we ultimately selected 15 participants’ data for further analysis.

For each individual’s R-fNIRS data, we visually inspected all the R-fNIRS time courses and found that there were unstable signals in some initial time points of the R-fNIRS scan for several participants, which could be attributable to the inadaptation of the subjects to the scanning environments and/or the unachieved stationary state of the scanning equipment. To obtain relatively steady signals and rule out the potential effects of unstable signals on subsequent functional connectivity and network topology analyses, we discarded the first 2 min data for each participant. The critical 2 min time point was chosen to ensure the steady of all time courses from each channel and participant. This procedure is employed in previous fNIRS studies [34]–[36]. We then digitally band-pass filtered (0.009 – 0.08 Hz) the raw optical density data to reduce the effects of low-frequency drift and high-frequency neurophysiological noise [22], [37]. Based on the filtered data from the two wavelengths (690 and 830 nm), we calculated the relative changes in the concentrations of oxy-Hb and deoxy-Hb with the modified Beer-Lambert law with a differential path length factor of 6 for each wavelength [38], [39]. Note that the sum of oxy-Hb and deoxy-Hb is defined as total-Hb. In this study, we chose oxy-Hb signal to perform comprehensive network analysis and evaluate reproducibility of network metrics across subjects and over time. Meantime, as a complement to hemoglobin contrasts, deoxy-Hb and total-Hb were also chosen to investigate whether they have similar network properties to those of oxy-Hb. The sampling rate for the optical signal was set to 25 Hz, which resulted in 12000 sample points from each 8 min dataset that could be used for further analysis.

### Construction of fNIRS-based Brain Networks

Nodes and edges are two fundamental elements of a network. In this study, the nodes were defined naturally as measurement channels and edges were defined as functional connectivity between nodes. Functional connectivity was quantified by computing Pearson correlation coefficients for the time series between pairs of nodes. Therefore, for each participant we obtained an *N*×*N* correlation matrix (*N* = 46, the number of fNIRS channels). We then averaged all of the individual correlation matrices and converted the resultant population-based mean correlation matrix into a binary graph (i.e., adjacency matrix) by applying a predefined correlation threshold, *T*, such that edges with absolute connectivity strengths, 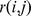, larger than *T* were set to 1 and all others were set to 0:
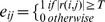
(1)


In this study, the correlation threshold *T* was determined in terms of sparsity (*S*) measure that is defined as the number of actual edges in a network divided by the maximum possible number of edges in a network. Because there is currently limited knowledge about R-fNIRS-based network topology, in this study, we thresholded the mean correlation matrix over the full sparsity range of 0<*S*<1 (interval = 0.01), which enables us to study network behaviors as a function of sparsity level. Figure 1 illustrates the schematic representation of network constructions using R-fNIRS.

### Network Analysis

We analyzed the topological properties of the group-level functional brain network derived from R-fNIRS data in terms of 8 global and 3 local nodal network metrics. The eight global network metrics included small-world properties (clustering coefficient, 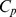, characteristic path length, 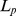, normalized clustering coefficient, 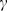, and normalized characteristic path length, 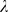), efficiency parameters (global efficiency, 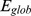, and local efficiency, 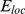), hierarchy (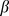), and modularity (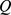). These metrics were used to characterize global topological organization of the whole-brain network. The 3 nodal metrics included nodal degree (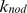), nodal efficiency (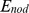), and nodal betweenness (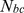), which were used to examine the regional characteristics of the functional brain network. In the Text S1, we briefly illustrated these metrics with a graph (or network) G consisting of N nodes and K edges. For more details about graph metrics, see [40].

### Statistical Analysis

To determine whether a network possesses small-worldness, hierarchy and modularity, the small-world parameters 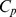 and 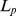, network efficiency 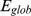 and 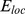, hierarchy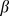 and modularity 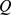 were compared to corresponding indices derived from 1000 comparable random null networks. The random networks were generated by preserving the same numbers of nodes and edges and the same degree distribution as the real brain network [41], [42]. Then a z-score was calculated as follows: 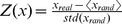, where *x* is a network parameter (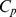,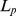,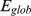,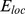,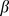 or 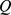) that has a value 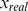 for the real brain network and has a mean 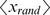 and standard deviation 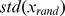 for 1000 random networks. A two-tailed significance level of 0.05 (z-score<−1.96 or z-score >1.96) was used to determine whether the real brain network possesses significantly non-random architecture.

### Reproducibility of Network Metrics

To determine the reproducibility of the network characteristics derived from R-fNIRS data, we implemented two additional complementary analyses. First, we divided the 15 participants into two independent subgroups (subgroup 1: n = 7; subgroup2: n = 8). There were no significant differences in age or gender between the two subgroups. Split-half analysis allows us to evaluate the reproducibility of network properties across participants. Second, we divided each participant’s whole 8-min dataset into two non-overlapping continuous 4-min sub-datasets (sub-dataset 1 and sub-dataset 2), leaving 6,000 data points for each participant in each sub-dataset. This division allows us to evaluate the reproducibility of network properties over time. For the both reproducibility analyses, functional brain networks were constructed and analyzed separately for each subgroup and each sub-dataset using the procedures as described above.

## Results

### Small-worldness and Efficiency

**Figure 2:**
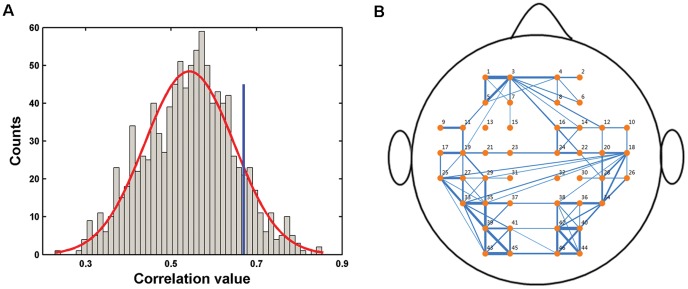
Characteristics of oxy-Hb-based group-level correlation network. (A) The correlation distribution and (B) its connectivity pattern. Only the topmost ranked 10% connection with correlation larger than 0.67 (blue line in A) are showed in B.

**Figure 3:**
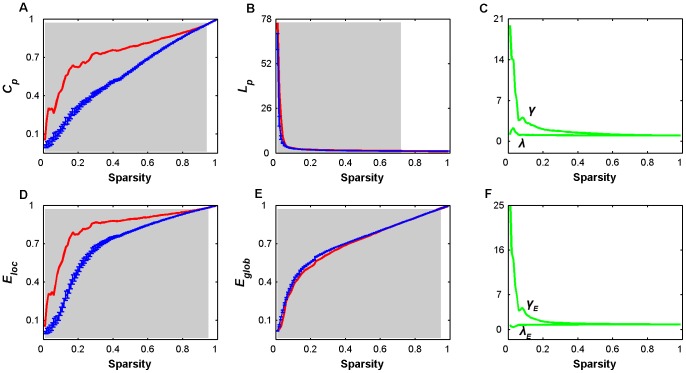
Small-world properties and network efficiency of oxy-Hb-based functional networks as a function of sparsity threshold. (A) Clustering coefficient, *C_p_*; (B) characteristic path length, *L_p_*; (C) normalized clustering coefficients,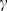, and normalized characteristic path lengths, 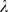; (D) local efficiency, *E_loc_*; (E) global efficiency, *E_glob_*; and (F) normalized local efficiency,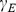, and normalized global efficiency, 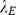. For a wide range of sparsity thresholds, the real brain networks have larger values of *C_p_* and *E_loc_* as than random networks; however, the values of *L_p_* and *E_glob_* are approximately equal, resulting in γ>1 and ∼1 as well as 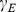
>1 and ∼1. These findings imply that R-fNIRS-based functional brain networks have prominent small-world features and are efficient in information processing. Error bars (A, B, D and E) correspond to standard errors of the mean for 1000 comparable random null networks. The gray areas indicate the sparsity range over which the parameters derived from real brain network are significantly (*P*<0.05) different from those derived from comparable random networks.

Using R-fNIRS data, we obtained a mean population-level correlation matrix (Fig. 1) and investigated its network topological attributes. Before presenting network topological results, we showed the distribution of correlation values within the matrix (Fig. 2A) and plotted the connectivity pattern for the topmost ranked 10% connections (correlation values >0.67) in anatomical space (Fig. 2B). We found that the correlation values showed an approximately normal distribution (mean = 0.54) and the connections were positioned in a structured manner. For subsequent network analysis, the mean correlation matrix was thresholded into a series of brain networks over the whole sparsity range of 0<*S*<1. Figure 3 shows the profiles of small-world parameters (clustering coefficient and characteristic path length) and network efficiencies (local efficiency and global efficiency) as functions of sparsity threshold. For both the real brain network and random networks, we found that the clustering coefficients (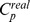
and 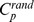) increased with sparsity threshold and that the characteristic path lengths (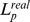
and 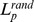) decreased monotonically with sparsity threshold (Fig. 3A and B). When compared to matched random networks, the clustering coefficients 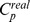 of the real brain network were larger than those 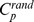
of random networks over a sparsity range of 0.01<*S*<0.93 (mean z-score = 22.01±10.95); however, the characteristic path lengths 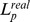 of the real brain network were larger than (but numerically approximate to) those 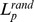
of their matched random networks over a sparsity range of 0.01<*S*<0.71 (mean z-score = 6.42×10^9^±2.38×10^10^) (Fig. 3A and B). These patterns resulted in normalized clustering coefficients 
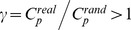
 and normalized characteristic path lengths 
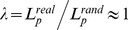
 (Fig. 3C), which are typical features of small-world topology.

For efficiency measures, local (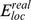 and 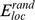) and global (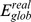 and 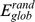) efficiency values that were derived from both the real brain network and matched random networks monotonically increased as a function of sparsity. Nevertheless, the real brain network exhibited higher local (0.01<*S*<0.94, mean z-score = 17.53±10.04) and lower (but numerically approximate) global efficiency (0.01<*S*<0.71, mean z-score = −9.82×10^9^±4.55×10^10^) in comparison to the random networks (Fig. 3D and E). This result generated a greater-than-1 normalized local efficiency 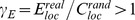 and an approximate equal-to-1 normalized global efficiency 
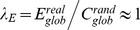
 (Fig. 3F). These findings suggest that relative to random networks, the real brain network is approximately equally efficient in distributed information processing but are more efficient in local information processing.

### Hierarchy

**Figure 4:**
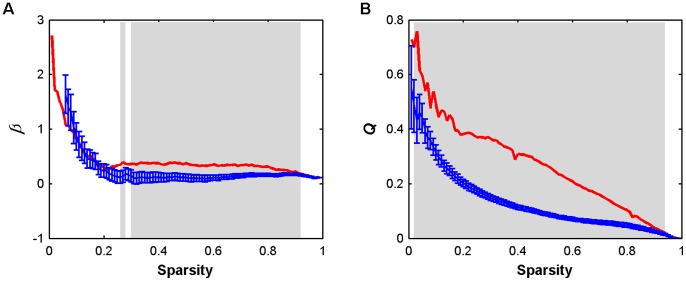
Hierarchy and modularity of oxy-Hb-based functional networks as a function of sparsity threshold. (A) Hierarchy coefficients,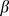, and (B) modularity, *Q*, of the real (red) and random (green) networks as functions of sparsity threshold. Error bars correspond to standard errors of the mean for 1000 comparable random null networks. The gray areas indicate the sparsity range over which the parameters derived from real brain network are significantly (*P*<0.05) different from those derived from comparable random networks. These results demonstrate a significant non-random hierarchical and modular organization of the fNIRS-based functional human brain networks.

The hierarchy coefficient curve had a profile that was characterized by an initial sharp drop, followed by a relatively steady state, and finally a gentle decline with increases in sparsity (sparsity cutoffs were 18% and 80%). When compared to random networks, the real functional brain network exhibited significantly larger hierarchy coefficients over a sparsity range of 0.30<*S*<0.91, with the most significant deviation at *S* = 0.74 (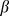 = 0.35, z-score = 6.55) (Fig. 4A). Among sparsity values of 0.30<*S*<0.91, the mean hierarchy coefficient was 0.33±0.05 and the corresponding mean z-score was 4.10±1.11. These results demonstrate a significant non-random hierarchical organization of the functional human brain networks that were based on fNIRS data.

### Modularity

The modularity derived from both the real brain network and random networks decreased monotonically with sparsity threshold (Fig. 4B). Nevertheless, the real brain network showed significantly non-random modular structure over almost the whole sparsity range (0.02<*S*<0.92) in comparison to random networks. The mean modularity value over the modular regime (i.e., 0.02<*S*<0.92) was 0.28±0.16 and the corresponding mean z-score was 15.64±8.75 (Fig. 4B). These results demonstrate a significant non-random modular organization of fNIRS-based functional human brain networks.

**Figure 5:**
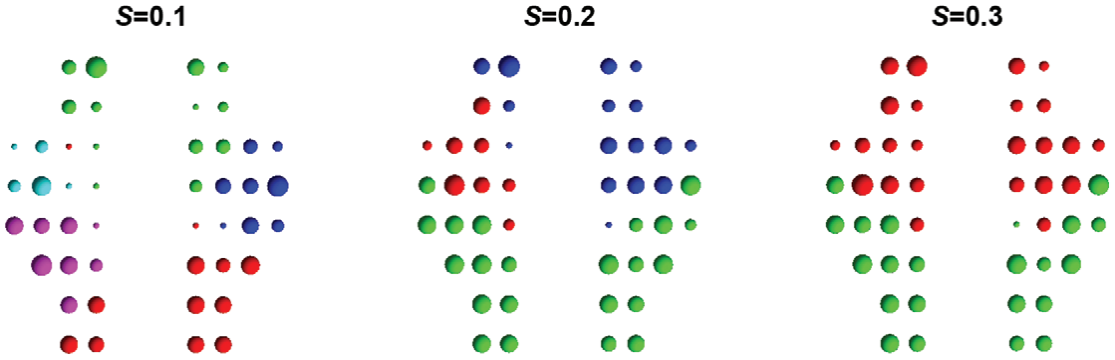
Modular architectures of oxy-Hb-based functional network at several selective sparsity thresholds (0.1, 0.2 and 0.3). Five, three and two functional modules are separately identified in the functional brain network. The size of each node denotes its relative nodal degree value in the brain network.

To further explore the refined modular architecture, we visualized the modular structure at *S* = 0.1, 0.2 and 0.3 (Fig. 5). At *S* = 0.1, five functional modules were identified at the maximum network modularity (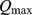 = 0.49, z-score = 6.32). Each of these modules was assigned a different color and was labeled from I to V as shown in Fig. 5A. Module I (green) consists of 13 nodes that are mostly from bilateral superior prefrontal and middle frontal areas that are known to be primarily involved in strategic/executive functions [43]. Module II (red) consists of 12 nodes and mostly includes regions from the occipital areas that are specialized for visual processing. Module III (blue) includes 8 nodes from right temporal and inferior frontal cortical areas and was therefore designated the right auditory/language module [44]. Module IV (pink) is composed of 8 nodes from motor and parietal cortices that are mainly associated with sensorimotor/spatial functions. Module V (cyan) includes 5 nodes from inferior frontal cortex and motor areas. Additionally, we found that these modules merged into larger clusters as sparsity values increased (Fig. 5B and C); this merging indicates a greater number of inter-module connections in the networks. Notably, the identified modules tended to cover bilateral homologous regions as sparsity values increased.

### Network Hubs

**Figure 6:**
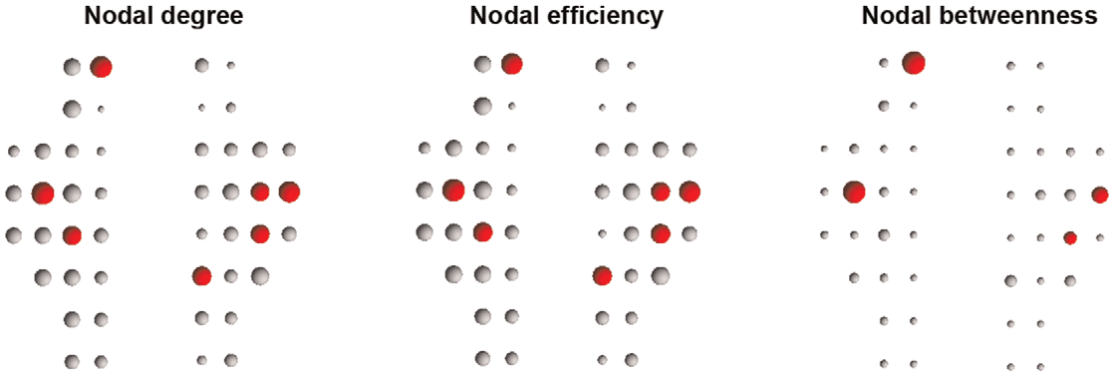
Hubs (red) in oxy-Hb-based functional network. The node sizes indicate their relative nodal centralities normalized to the corresponding mean for all nodes in the network. A region was considered a hub if its normalized nodal centrality was at least 1 standard deviation greater than the mean of all the nodes in the network.

In this study, we identified network hubs according to three different regional nodal parameters: degree, 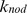, efficiency, 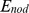 and betweenness centrality, 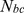. A node was considered a hub if any one of the three nodal metrics was at least 1 standard deviation greater than the average of all nodes in the network. Notably, functional brain networks were constructed over a series of continuous sparsity threshold (0<*S*<1) in the current work, therefore each of the three nodal metrics was a function of sparsity. To simplify analysis, for each node we calculated a threshold-independent scalar by calculating the area under curve (over sparsity) for each nodal metric of degree, efficiency and betweenness. These scalars were used to identify hub regions. Sorting in terms of 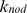, we identified seven hub nodes in the network (Fig. 6A), which were predominately located in frontal (ch3, ch18, ch19 and ch20), parietal (ch38) and temporal cortices (ch28 and ch29). These seven regions were also identified as hubs according to calculations of nodal efficiency (Fig. 6B). Four out of these seven nodes (ch3, ch18, ch19 and ch28) were also identified as hubs based on nodal betweenness (Fig. 6C).

### Reproducibility of Our Results

#### Reproducibility across subjects

**Figure 7:**
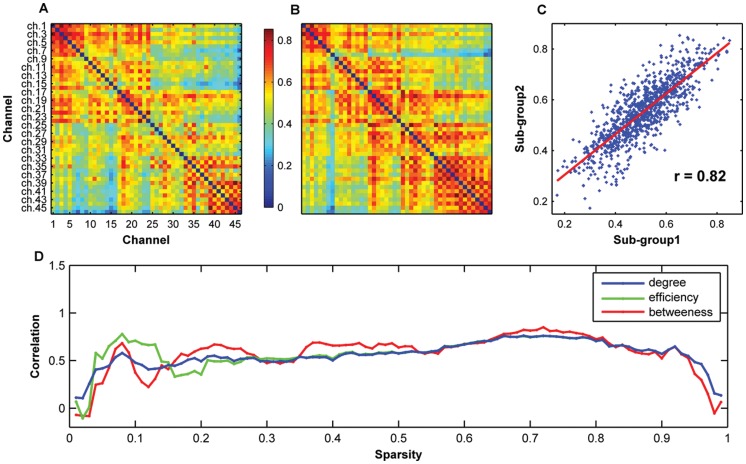
Reproducibility of oxy-Hb-based functional network properties across subjects. The mean correlation matrices derived from subgroup 1 (A) and subgroup 2 (B) are highly similar to each other (C). The regional nodal metrics also exhibited strong correlations between the two subgroups (D) over a wide range of sparsity thresholds (degree: r = 0.56±0.14; efficiency: r = 0.57±0.16; and betweenness: r = 0.57±0.20). These results suggest a high reproducibility of fNIRS-based brain network metrics across subjects.

We divided all of the participants into two non-overlapping subgroups to study the reproducibility of network metrics across subjects. We found that the mean correlation matrices were very similar between the two subgroups as shown by both qualitative visual inspection (Fig. 7A and B) and quantitative spatial correlation analysis (r = 0.82, *P* = 0.00) (Fig. 7C). The regional nodal metrics between the two subgroups also exhibited strong correlations over a wide range of sparsity values (degree: r = 0.56±0.14; efficiency: r = 0.57±0.16; and betweenness: r = 0.57±0.20) (Fig. 7D). These results suggest that fNIRS-based brain network metrics are stable across subjects. Notably, the variability of nodal betweenness was relatively larger as compared to the other two nodal metrics.

#### Reproducibility over time

**Figure 8:**
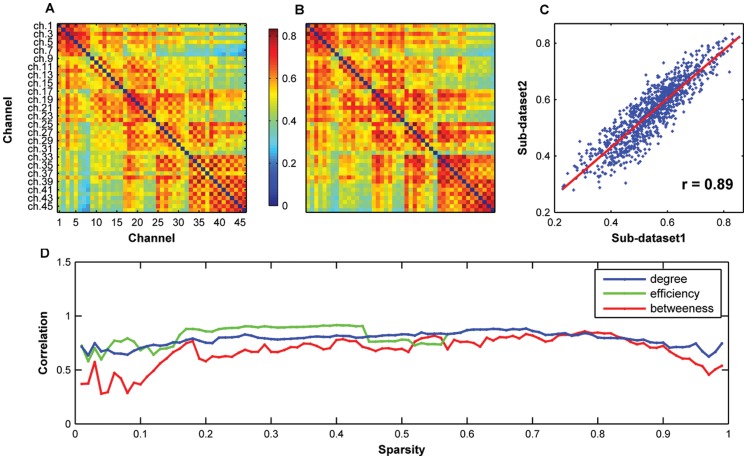
Reproducibility of oxy-Hb-based functional network properties over time. The mean correlation matrices derived from sub-dataset 1 (A) and sub-dataset 1 (B) are highly similar to each other (C). The regional nodal metrics also exhibited strong correlations between the two sub-datasets (D) over a wide range of sparsity thresholds (degree: r = 0.79±0.06; efficiency: r = 0.81±0.08; and betweenness: r = 0.68±0.14). These results suggest a high reproducibility of fNIRS-based brain network metrics over time.

We divided the whole dataset into two equal parts, each containing 6000 time points, and studied the reproducibility of network metrics over time. We found that the mean correlation matrices from sub-dataset 1 (the first 4 min data) and sub-dataset 2 (the last 4 min data) were highly resemblant (r = 0.89, *P* = 0.00) (Fig. 8A, B and C). Moreover, the regional nodal metrics between the two sub-datasets also showed strong correlations over a wide range of sparsity values (degree: r = 0.79±0.06; efficiency: r = 0.81±0.08; and betweenness: r = 0.68±0.14) (Fig. 8D). These results suggest that fNIRS-based brain network metrics are highly reproducible over time. Again, nodal betweenness showed a relative lower reproducibility and higher variance as compared to the other two nodal metrics.

### Network Properties Derived from Deoxy-Hb and Total-Hb

**Figure 9:**
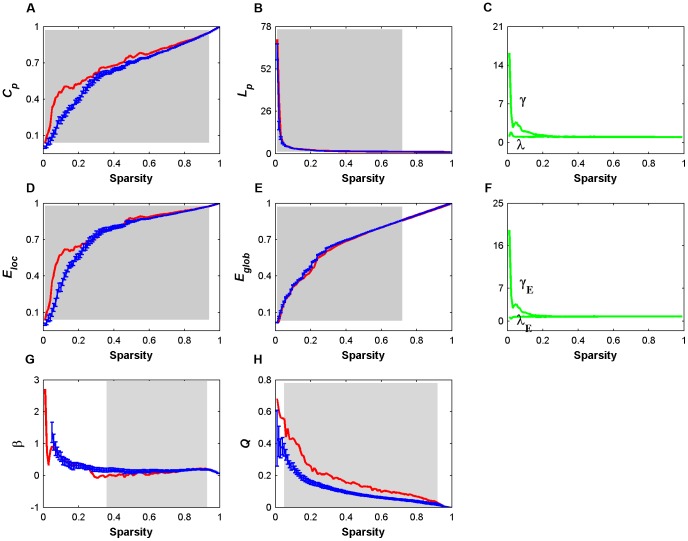
Small-world properties, network efficiency, Hierarchy (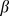), and modularity (*Q*) of deoxy-Hb-based functional networks as a function of sparsity threshold. Error bars correspond to standard deviation of the mean for 1000 comparable random null networks (blue lines). The gray areas indicate the sparsity range over which the parameters derived from real brain network (red lines) are significantly (*P*<0.05) different from those derived from comparable random networks. These results demonstrate efficient small-world properties, significant non-random hierarchical and modular organization of deoxy-Hb-based functional brain networks.

**Figure 10:**
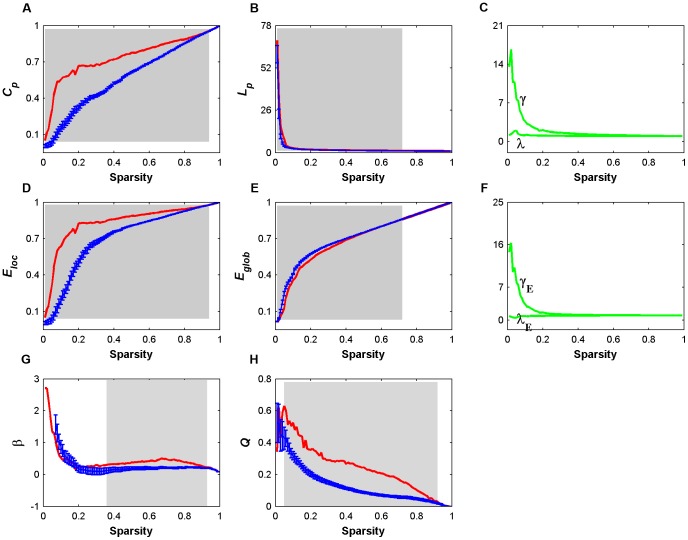
Small-world properties, network efficiency, Hierarchy (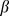), and modularity (*Q*) of total-Hb-based functional networks as a function of sparsity threshold. Error bars correspond to standard deviation of the mean for 1000 comparable random null networks (blue lines). The gray areas indicate the sparsity range over which the parameters derived from real brain network (red lines) are significantly (*P*<0.05) different from those derived from comparable random networks. Again, these results demonstrate efficient small-world properties, significant non-random hierarchical and modular organization of total-Hb-based functional brain networks.

**Figure 11:**
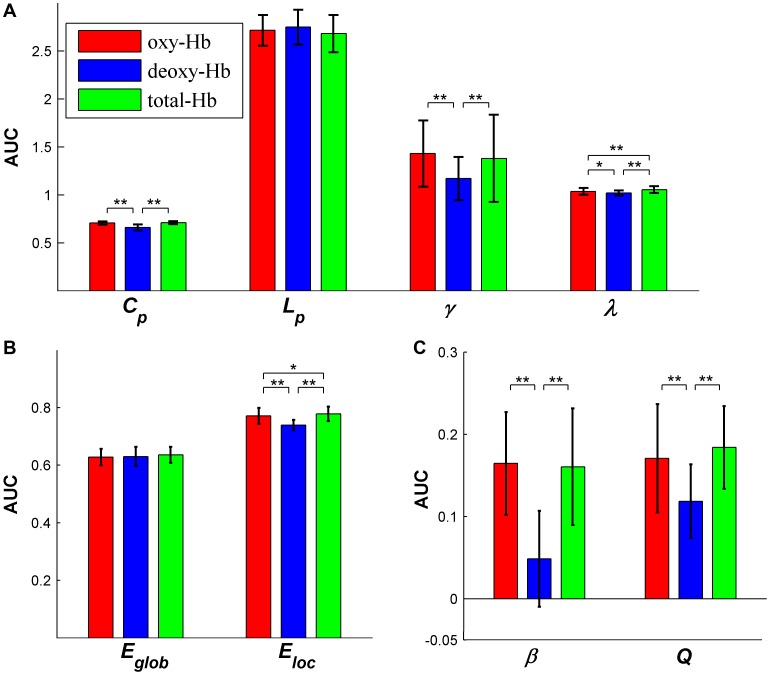
The differences of network properties derived from oxy-Hb, deoxy-Hb and total-Hb signals. Bars show the mean areas under curves (AUC) of (A) small-world parameters (*C_p_*, *Lp,* 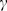 and 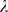), (B) network efficiency (*E_loc_* and *E_glob_*), (C) hierarchy coefficient (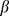) and modularity (*Q*). Error bars correspond to standard deviation of the mean across participants. The asterisk indicates *P*<0.05 and double asterisk indicates *P*<0.01.

The construction and analysis of R-fNIRS brain network were also repeated with deoxy-Hb and total-Hb contrasts. It is noticed that most network metrics derived from these two signals exhibited similar profiles to those of oxy-Hb such as 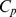,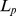,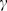,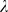,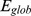,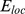,
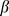
_,_ and 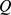. Moreover, these metrics differed significantly from those of comparable random networks, indicating non-random features of efficient small-worldness, hierarchy and modularity (Figs .9 and 10). To test whether there exists significant differences in network parameters derived from oxy-Hb, deoxy-Hb and total-Hb contrasts, we calculated a threshold-independent scalar of area under the curve (AUC, i.e., the integral) for each network metric under each hemoglobin signal and performed one-way repeated-measure ANOVA (across subjects) on these scalars. The results showed that 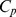,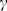,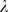, 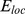,
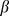
_,_ and 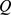 differed significantly (*P*<0.05) among the three hemoglobin contrasts. Further post-hoc comparisons (paired t-tests) revealed that compared with deoxy-Hb-based networks, oxy-Hb- and total-Hb-based networks had greater 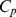 (oxy-Hb > deoxy-Hb: *P* = 2.44e^−5^; total-Hb > deoxy-Hb: *P* = 1.70e^−5^), 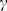
(oxy-Hb > deoxy-Hb: *P* = 1.0e^−3^; total-Hb > deoxy-Hb: *P* = 8.71e^−4^), 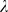
(oxy-Hb > deoxy-Hb: *P* = 0.034; total-Hb > deoxy-Hb: *P* = 0.0026), 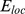
(oxy-Hb > deoxy-Hb: *P* = 6.78e^−5^; total-Hb > deoxy-Hb: *P* = 3.14e^−5^), 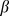 (oxy-Hb > deoxy-Hb: *P* = 2.36e^−5^; total-Hb > deoxy-Hb: *P* = 3.2e^−5^), and 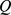 (oxy-Hb > deoxy-Hb: *P* = 0.011; total-Hb > deoxy-Hb: *P* = 0.0013). Meantime, we also found that compared with oxy-Hb-based networks, total-Hb-based networks had greater values in 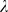 (total-Hb > oxy-Hb: *P* = 0.0037) and 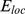 (total-Hb > oxy-Hb: *P* = 0.017) (Fig. 11).

## Discussion

In the present study, we investigated the topological properties of functional networks of the human brain using R-fNIRS and graph-theory methods. We found that the R-fNIRS-based functional brain network exhibited: 1) an optimal small-world configuration for both localized and distributed information processing; 2) a hierarchical organization that supports top-down relationships between nodes and minimizes wiring costs; 3) a modular architecture where the identified modules correspond to several well-known brain functions; and 4) heterogeneous nodal centrality of core hubs. The findings were highly reproducible across participants and over time. Collectively, we demonstrate that human brain functional networks can be constructed from R-fNIRS data and the connectivity networks are specially organized in the light of several non-trivial wiring principles.

**Figure 12:**
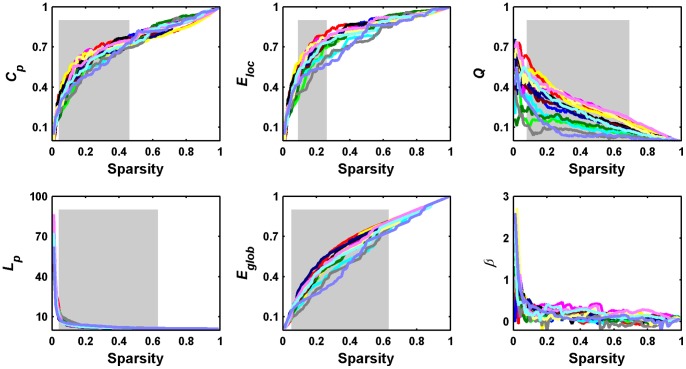
Small-world properties, network efficiency, hierarchy (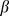), and modularity (*Q*) of individual oxy-Hb-based functional networks as a function of sparsity threshold. The gray areas indicate the sparsity range over which the parameters derived from all individual brain networks are significantly (*P*<0.05) different from those derived from comparable random networks.

Utilizing R-fNIRS we investigated the topological organization of functional brain network at a group level. Functional brain networks can be constructed and further studied at both individual level [45]–[48] and group level [11], [49]–[51]. Here, we aim to systematically characterize topological organization of a population-based representative functional brain network capturing the common connectivity pattern across population (i.e., backbone), rather than a subject-specific and very detailed network for an entire individual brain. Furthermore, a previous study indicated that for R-fNIRS data group-level functional connectivity analyses have better test-retest reliability in comparison with subject-level analyses [52]. Nevertheless, studying network topology at group level will inevitably neglect information of inter-subject variances that are potentially meaningful [53]. Therefore, we reanalyzed our data individually and the results showed that all individual networks exhibited small-worldness, modularity and hierarchy (Fig. 12). Meanwhile, we also noted obvious inter-individual variances in these network metrics (Fig. 12) that may be related with individual traits. To better understand these variances, future studies on the relationship between network topology and behavior/cognitive ability across individuals will provide insights into this issue.

We found that the population-based functional brain network derived from R-fNIRS data exhibited small-world architecture, which enables both local interconnectivity or cliquishness and global overall routing simultaneously [54], [55]. Small-worldness is a ubiquitous characteristic of complex systems [56], including the human brain [57]. Utilizing resting-state fMRI data, previous studies also demonstrated the small-wordness in functional brain networks across populations [49], [50]. For example, Salvador and colleagues found that the local clustering coefficient of group-level brain network was approximately two times greater than those of random networks, whereas the mean shortest path length between any two brain regions was approximately equivalent to the random network [49]. Here we constructed functional brain networks using novel R-fNIRS and also observed small-world topology, suggesting that the small-worldness is a universal principle for functional wiring of the human brain regardless of the distinct mechanisms of different imaging techniques. The results provide further support for the notion that the human brain is naturally organized in an optimal fashion for maximizing the efficiency of information processing and minimizing required wiring costs. However, it should be noted that a directly quantitative comparison between the current study and those abovementioned fMRI studies is unsuitable because of different network sizes (46 in the current study vs. 45 in [49] vs. 90 in [50]) and connectivity measures (Pearson correlation in the current study vs. partial correlation [49] vs. wavelet correlation [50]) employed in these studies, all of which can affect network topology [47], [58]–[60]. Further work is needed to quantitatively characterize the relationship of network topology between these two modalities by simultaneously recording resting-state fNIRS and fMRI signals [61].

In addition to small-world topology, the R-fNIRS-based functional brain networks also showed hierarchal and modular structures. Hierarchical organization refers to a configuration in which small groups of nodes are organized into increasingly large groups in a hierarchical manner [62]. This organization gives a network non-trivial features that support top-down relationships and minimize wiring costs but is vulnerable to attacks on hubs. Based on inter-regional covariation of gray matter volume in MRI data, Bassett and colleagues reported significant levels of hierarchical organization in anatomical human brain networks [63]. As for functional brain networks, non-random hierarchical architectures are also reported based on R-fMRI data [59], [64]. These findings suggest that hierarchal organization is a general principle governing the interconnected patterns of both structural and functional human brain networks. Here, using R-fNIRS data our findings provide new evidence in support of the generality of hierarchical organization in human brain networks. Numerous studies have found evidence of modular architecture in the anatomical and functional human brain networks [65]. Modular structure in a network has the advantage of allowing evolutionary or developmental optimization of one functional module without risking a loss of function in other modules and has been proven to be an efficient solution to multiple selection pressures on network evolution [66]. Using the novel R-fNIRS, we also detected evidence of modular topology in human brain networks with functionally related structures in the same modules (e.g., visual module).These results lend support to the notion of modularity as a fundamental design principle in brain networks of the human brain. However, we note that the modularity derived from R-fNIRS-based brain networks here seems to be smaller than that of population-based brain networks derived from R-fMRI data [11] (0.49 vs. 0.66) at approximately comparable sparsity. These differences could be due to the different network sizes or different temporal resolutions (46 vs. 90).

Beyond global organization metrics of small-worldness, hierarchy and modularity, we also investigated local nodal centrality. Nodes with high centrality (i.e., hubs) play pivotal roles in controlling the information flow by serving as critical gateways for the integration of diverse informational sources, and they balance the opposing pressure to evolve segregated, specialized pathways. Hubs also help to minimize wiring and metabolism costs by providing a limited number of long-distance connections that integrate local networks [67]. In the present study, we used three nodal measures to characterize nodal centrality from different perspectives and to comprehensively identify hubs in the R-fNIRS-based functional brain network. Four regions that were predominately located in frontal and temporal regions were consistently identified as hubs. Using other neuroimaging techniques (e.g., diffusion tensor imaging, structural MRI and resting-state fMRI), these regions have also been proven to serve as hubs in structural and/or functional brain networks [50], [68], [69]. Nonetheless, we noted that several commonly identified hub regions previously (e.g., posterior parietal regions) were not detected in the current study. This may be due to the limited coverage and spatial localization of R-fNIRS. Together, despite of some inconsistencies, hub phenomenon (heterogeneous nodal centrality) is another universal feature of human brain networks.

To evaluate the robustness of the entire network features studied above, we performed split-half analyses to test the reproducibility of our results across subjects and over time. The results revealed that the R-fNIRS-based functional brain networks were highly reproducible across subjects and stable over time. These encouraging results suggest that R-fNIRS could be considered as a promising and reliable technique for the study of topological properties of functional brain networks. However, further methodological studies are needed, such as studies of test-retest reliability.

The potential usefulness of fNIRS in brain network studies could be attributed to its unique advantages such as safety, portability, technical simplicity, and low cost relative to the costs of other imaging techniques, such as fMRI. More importantly, fNIRS can be performed in a quiet environment and can accommodate measurements from a person in a sitting position without physical restraint, which makes it an ideal choice for studying members of special populations who are not suited for participation in fMRI studies (e.g., neonates and infants) or people who suffer from severe movement disorders (e.g., patients with attention deficit hyperactivity disorder). Moreover, fNIRS has a high temporal sampling rate (typically ∼10 Hz, 25 Hz in our study) and thus can avoid signal bias due to high-frequency cardiac or respiratory noise or low-frequency fluctuations in the hemodynamic signal. Therefore, it provides a promising technique for measuring neural activity. Last but not least, fNIRS provides a comprehensive assessment of hemodynamics and metabolism by measuring the changes in oxy-Hb, deoxy-Hb and total-Hb. These hemoglobin species have distinct relationships to the neuronal or vascular origin. Nevertheless, we show that all the networks derived from the three hemodynamic contrasts consistently display several non-random features, including efficient small-worldness, hierarchy and modularity. These findings strongly suggest that fNIRS can offer unique opportunities to exploit organizational principles governing functional brain networks. However, it should be noted that there also exists significant differences in quantitative network topology among the three contrasts (Fig. 11). The discrepancy could be attributed to distinct relationships to neuronal or vascular origin among the three hemoglobin species. Future studies are requested to determine the origin of these discrepancies and to study specificity of different contrasts in revealing condition- or disease-related effects on topological architecture of the brain. Collectively, given the aforementioned advantages of fNIRS and the reproducible and reliable hallmarks of fNIRS in characterizing brain network organization that have been demonstrated in the current study, we suggest that fNIRS be used to study system-level brain network architecture in people with conditions of development or various neurological and psychiatric disorders, for whom other imaging techniques are not suitable.

There are several limitations of fNIRS-based studies of brain networks. Frist, fNIRS has a spatial resolution on the order of 10 to 20 mm, which is lower than the spatial resolution of fMRI (1 to 3 mm). Limited spatial resolution may directly result in coarse spatial localization of brain regions. Thus, it is difficult to accurately locate the corresponding functional brain area for each network node. Moreover, the number of probes (sources and detectors) is limited in most fNIRS systems, which makes it difficult to apply fNIRS for whole-brain network studies and further inhibits the exploration of topological connectivity among multiple cortical neural systems. Second, current methodology in processing fNIRS signals is far from perfect, especially in denoising (e.g, motion artifacts). Therefore, potential noises may contaminate accurate characterization of network topology. Last but not least, fNIRS is limited in its depth of penetration, so it is impossible to investigate the functional connectivity of deeper cortical structures such as the thalamus and caudate nucleus. These limitations are considered to be open and important issues in the development of fNIRS investigation techniques.

### Conclusions

In summary, we used R-fNIRS to study the topological organization of functional networks in the human brain and observed several predominant principles underlying its wiring, including small-worldness, hierarchy, modularity and hubs. The current study presents a methodological framework for R-fNIRS in performing functional brain network studies that could be further extended to explore normal development, aging, and various diseases, such as stroke in infants, from an integration perspective.

## Supporting Information

Text S1 A description of network metrics.
(DOCX)
