# Correction: Revealing Topological Organization of Human Brain Functional Networks with Resting-State Functional Near Infrared Spectroscopy

**DOI:** 10.1371/annotation/8941aee3-4bb8-42a0-b09a-e7c416beeef7

**Published:** 2013-01-22

**Authors:** Haijing Niu, Jinhui Wang, Tengda Zhao, Ni Shu, Yong He

There was a formatting error in the title of the article. The word "near" should have been capitalized. The correct title is: Revealing Topological Organization of Human Brain Functional Networks with Resting-State Functional Near Infrared Spectroscopy.

The correct citation is: Niu H, Wang J, Zhao T, Shu N, He Y (2012) Revealing Topological Organization of Human Brain Functional Networks with Resting-State Functional Near Infrared Spectroscopy. PLoS ONE 7(9): e45771. doi:10.1371/journal.pone.0045771

