# Supplementary material for: Revealing Topological Organization of Human Brain Functional Networks with Resting-State Functional near Infrared Spectroscopy
Source: PLoS One. 2012 Sep 24;7(9):e45771. doi: 10.1371/journal.pone.0045771 (PMC3454388; doi:10.1371/journal.pone.0045771)
Supplement: Text S1 — A description of network metrics. (DOCX) [file pone.0045771.s001.docx]

**Supporting Information**

We illustrated the network metrics using a graph (or network) G that consisted of N nodes and K edges. For the details of these network definitions, see [[1](#_ENREF_70),[2](#_ENREF_71)].

***Global network metrics***

*Small-world parameters*

The clustering coefficient $C_{p}$ of a network G is defined as the average of the clustering coefficients of all nodes in the network, where the nodal clustering coefficient for a given node $i$ is calculated as [[3](#_ENREF_54)]:

$C_{pnod}\left( i \right)=\frac{2N_{i}}{k_{nod}\left( i \right)\left( k_{nod}\left( i \right)-1 \right)} ,$ (2)

where $N_{i}$ denotes the number of existing connections among the neighbors of node $i$, and $k_{nod}$ represents the number of edges that are connected to node $i$. The cluster coefficient reflects the local interconnectivity and cliquishness of a network.

The characteristic path length $L_{p}$ of a network G is defined as the average of the shortest path lengths between all pairs of nodes in the network G [[3](#_ENREF_54)]:

$L_{p}=\frac{1}{N(N-1)}\sum_{i\neq j\in G} d_{ij} ,$ (3)

where $d_{ij}$ is the shortest path length between node $i$ and node $j$. The shortest path length was the minimum number of edges included in the path connecting these two nodes. The characteristic path length quantifies the mean separation or overall routing efficiency of a network [[4](#_ENREF_55)]. To deal with the possible dilemma of isolated or disconnected nodes, we calculated the characteristic path length as the harmonic mean of the shortest path lengths in actual operation [[5](#_ENREF_72)].

To examine the small-world properties of a network, the normalized clustering coefficient $\gamma={C_{p}^{real}}/{C_{p}^{rand}}$and the normalized characteristic path length $\lambda={L_{p}^{real}}/{L_{p}^{rand}}$were computed [[3](#_ENREF_54)]. $C_{p}^{\mathrm{real}}$and $L_{p}^{\mathrm{real}}$ are the clustering coefficient and the characteristic path length of a real network, and $C_{p}^{\mathrm{rand}}$ and $L_{p}^{\mathrm{rand}}$ represent the means of corresponding indices derived from 1000 matched random networks that have the same numbers of nodes and edges, and the same degrees of distribution as the real networks [[6](#_ENREF_41),[7](#_ENREF_73)]. Typically, a small-world network should meet the following criteria: $\gamma>1$and$\lambda\approx1$.

*Network efficiency*

Network efficiency is a more biologically relevant metric to describe brain networks and can be defined at local and global levels to measure of the ability of a network to transmit information over a local subset of nodes and over the whole network, respectively. For network G, the global efficiency $E_{glob}$ is defined as the inverse of the harmonic mean of the shortest path length between any two nodes [[8](#_ENREF_74)]:

$E_{glob}=\frac{1}{N(N-1)}\sum_{i\neq j\in G} \frac{1}{d(i,j)}$ , (4)

where $d(i,j)$ is the shortest path length between node $i$ and node$j$. Note that the global efficiency is numerically reciprocal to the characteristic path length of a network [[8](#_ENREF_74)]. The local efficiency $E_{loc}$ of network G is defined as the average of the local efficiencies of all nodes where the local nodal efficiency for a given node $i$ is the global efficiency of the subgraph composed of the nearest neighbors of node$i$.

*Hierarchy*

Mathematically, the hierarchical structure of a network can be quantified by the $\beta$ coefficient, which is a parameter of the power-law relationship between nodal clustering, $C_{pnod}$, and the degree,$k_{nod}$ , of the nodes in a network [[9](#_ENREF_62)]:

$C_{pnod}\approx k_{nod}^{-\beta}$ , (5)

A large positive value of $\beta$ signifies typical hierarchical structure where highly connected nodes are connected predominantly to nodes that are not otherwise connected to each other. That is, the larger the degree, the lower the clustering coefficient. We estimated $\beta$ by fitting a linear regression line to the plot of *log*(C) versus *log* (k) for the network at any given sparsity.

*Modularity*

Modularity reflects the degree to which a network is organized into a modular or community structure. For a given partition $p$ of a network, the modularity is defined as [[10](#_ENREF_75)]:

$\mathcal{Q}\left( p \right)=\sum_{m-1}^{M} \left[ \frac{l_{m}}{L}-\left( \frac{d_{m}}{2L} \right)^{2} \right]$ , (6)

where M is the number of modules, $L$ is the number of connections in the network, $l_{m}$is the number of connections between nodes in module $m$ , and $d_{m}$is the sum of the degrees of the nodes in module $m$. The modularity, *Q*, quantifies the difference between the actual number of intra-module links and the expected number for the same partition in a randomized network. The objective of a modular detection algorithm is to find the partition $p$ that maximizes the modularity *Q*. In practice, a *Q* value above 0.3 is a good indicator of significant modules in a network [[11](#_ENREF_76)].

***Regional nodal metrics***

*Nodal degree*

The degree of a given node $i$ is defined as the number of edges linked to the node:

$k_{nod}\left( i \right)=\sum_{j\neq i\in G} a_{ij}$ , (7)

where $a_{ij}$is the $i^{th}$row and $j^{th}$column element in the formerly obtained adjacency matrix.

*Nodal efficiency*

The efficiency of a node $i$ is measured as [[12](#_ENREF_45)]:

$E_{nod}(i)=\frac{1}{N-1}\sum_{j\neq i\in G} \frac{1}{d(i,j)}$ , (8)

where $d(i,j)$ is the shortest path length between node $i$ and node $j$.

*Nodal betweenness*

Nodal betweenness quantifies how important a node is within a network. It is defined as [[13](#_ENREF_77)]:

$N_{bc}\left( i \right)=\sum_{m\neq i\neq n\in G} \frac{\delta_{mn}(i)}{\delta_{mn}} ,$ (9)

where $\delta_{mn}$ is the total number of shortest paths from node *m* to node *n* and $\delta_{mn}(i)$is the number of shortest paths from node $m$ to node $n$ that pass through node $i$. A node with high *N*_bc_ indicates large impacts of the node on the information flow over the whole network.

**References**

1. Boccaletti S, Latora V, Moreno Y, Chavez M, Hwang DU (2006) Complex networks: Structure and dynamics. Physics Reports 424: 175-308.

2. Rubinov M, Sporns O (2010) Complex network measures of brain connectivity: Uses and interpretations. NeuroImage 52: 1059-1069.

3. Watts DJ, Strogatz SH (1998) Collective dynamics of 'small-world' networks. Nature 393: 440-442.

4. Latora V, Marchiori M (2003) Economic small-world behavior in weighted networks. The European Physical Journal B - Condensed Matter and Complex Systems V32: 249-263.

5. Newman MEJ (2003) The Structure and Function of Complex Networks. SIAM Review 45: 167-256.

6. Maslov S, Sneppen K (2002) Specificity and Stability in Topology of Protein Networks. Science 296: 910-913.

7. Milo R, Shen-Orr S, Itzkovitz S, Kashtan N, Chklovskii D, et al. (2002) Network motifs: simple building blocks of complex networks. Science 298: 824-827.

8. Latora V, Marchiori M (2001) Efficient Behavior of Small-World Networks. Physical Review Letters 87: 198701.

9. Ravasz E, Barabasi AL (2003) Hierarchical organization in complex networks. Phys Rev E Stat Nonlin Soft Matter Phys 67: 026112.

10. Newman MEJ (2006) Modularity and community structure in networks. Proceedings of the National Academy of Sciences 103: 8577-8582.

11. Clauset A, Newman MEJ, Moore C (2004) Finding community structure in very large networks. Physical Review E 70: 066111.

12. Achard S, Bullmore E (2007) Efficiency and Cost of Economical Brain Functional Networks. Plos Computational Biology 3: e17.

13. Linton CF (1977) A Set of Measures of Centrality Based on Betweenness. Sociometry 40: 35-41.
